# Comparison of the Serological Reactivity of Lipopolysaccharides from Japanese and Western Strains of *Helicobacter pylori* to Sera from *H. pylori*-Positive Humans

**DOI:** 10.5402/2012/162816

**Published:** 2012-07-26

**Authors:** Ken-ichi Amano, Shin-ichi Yokota, Mario A. Monteiro

**Affiliations:** ^1^Bioscience Education and Research Center, Akita University, 1-1-1, Hondo, Akita 010-8543, Japan; ^2^Department of Microbiology, Sapporo Medical University School of Medicine, South-1, West-17, Chuo-ku, Sapporo 060-8556, Japan; ^3^Department of Chemistry, University of Guelph, Guelph, ON, Canada N1G 2W1

## Abstract

We compared the serological reactivity of lipopolysaccharides (LPS) isolated from Japanese and Western strains of *Helicobacter pylori* against anti-Lewis antigen monoclonal antibodies and *H. pylori*-positive Japanese sera. The two LPS from Western strains (26695 and O:2) did not react with any sera from Japanese patients, while all LPS from Japanese strains and the Sydney strain reacted with these sera. We propose that LPS of all Japanese smooth strains share either one of two epitopes, which are termed highly antigenic and weakly antigenic epitopes, present in the O-polysaccharide portion, and these epitopes are independent the Lewis antigens. The present findings indicated that the two Western strains lacked the two epitopes, which are shared by all Japanese strains.

## 1. Introduction

*Helicobacter pylori* is a gram-negative and microaerophilic bacterium that is recognized as a major cause of chronic gastritis, peptic ulcer, and gastric cancer [1, 2]. The chemistry and biology of *H. pylori *lipopolysaccharides (LPS) have been extensively studied. Aspinall et al. [3] and Monteiro et al. [4] determined the structures of the O-polysaccharides of *H. pylori* LPS and found them to be the same as the Lewis X (Le^x^) and Lewis Y (Le^y^) determinants of human cell-surface glycoconjugates. Appelmelk et al. [5] suggested that the mimicry of Lewis antigens by this bacterium raised titers of autoantibodies to Lewis antigens in infected individuals. However, we find no significant titers of anti-Lewis antigen antibodies in the sera of *H. pylori*-positive humans [6]. On the other hand, we have observed that all *H. pylori* smooth-type LPS possess either one of two antigenic epitopes (the highly antigenic and the weakly antigenic epitopes) in their polysaccharide regions [7–9]. These are unlikely to be related to the structures mimicking Lewis antigens. Most *H. pylori*-infected individuals have high titers of antibody to one of these antigenic epitope (the highly antigenic epitope). So we proposed that an LPS possessing this antigenic epitope would be a strong candidate for an antibody diagnosis of *H. pylori* infection [10]. Monteiro et al. [11] compared the structures between *H. pylori* LPS isolated from Asian and Western patients and found that the Asian strains showed a stronger tendency to produce type 1 blood groups. In this paper, we compared the reactivity of *H. pylori* LPS from Japanese and Western strains to the sera of *H. pylori*-positive humans.

## 2. Materials and Methods

### 2.1. Bacterial Strains and Preparation of LPS

Japanese *H. pylori* strains (GU2, DU1, CA2, CA4, and CA5) were isolated from biopsy specimens of lesions obtained from patients with gastric ulcer (GU), duodenal ulcer (DU), or gastric cancer (CA) in the Sapporo Medical University Hospital (Sapporo, Japan) as described previously [6]. Extraction and purification of LPS were described by Amano et al. [12]. Isolation of Western *H. pylori* strains [NCTC11637, Sydney (SS1), 26695, and O:2] and purification of LPS were as described by Monteiro et al. [4].

### 2.2. Human Sera

Sera from *H. pylori*-positive patients were donated by the Sapporo Medical University Hospital as previously described [7]. The *H. pylori* infection status of each individual was determined with the Determinar *H. pylori* antibody enzyme immunoassay kit (Kyowa Medicus, Tokyo, Japan). 

### 2.3. Antibodies and Immunoblotting

 Murine monoclonal antibodies (MAbs) against Lewis antigens used in the study were as follows: clone 73-30 [anti-Le^x^ immunoglobulin M (IgM) (Seikagaku Kogyo, Tokyo, Japan)], BG8 and BG6 [anti-Le^y^ IgM and anti-Le^b^ IgM, respectively (Signet Laboratories, Dedham, Mass, USA)], and MAB2108 [anti-Le^a^ IgG1 (Chemicon, Temecula, Calif, USA)]. Sodium dodecyl sulfate-polyacrylamide gel electrophoresis (SDS-PAGE) and immunoblotting were performed as described previously [6]. The LPS profile on the gel was developed by silver staining as described previously [6].

## 3. Results

**Figure 1:**
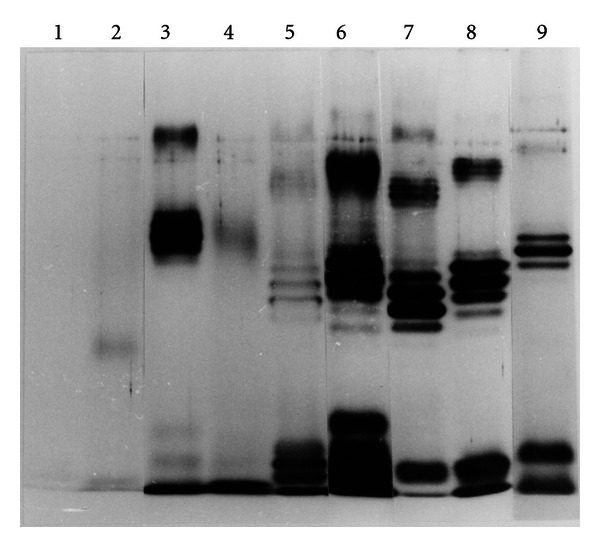
Profile of *H. pylori* LPS from Japanese and Western strains on a silver-stained SDS-PAGE gel. 1, NCTC11637-LPS; 2, Sydney strain-LPS; 3, 26695-LPS; 4, O:2-LPS, 5, GU2-LPS; 6, DU1-LPS; 7, CA2-LPS; 8, CA4-LPS; and 9, CA5-LPS.

 The molecular sizes and microheterogeneity of *H. pylori* LPS from Japanese and Western strains were compared on an SDS-PAGE gel after silver staining (Figure 1). LPS from all strains except NCTC11637 showed ladder bands, which are one of the characteristics of smooth-type LPS, in the high molecular weight area, and some bands characteristic of rough-type LPS in the low-molecular-weight area. LPS from NCTC11637 showed only one faint band in the fast migration zone of the gel but no ladder bands. The specificity of anti-Lewis antigen MAbs for *H. pylori* LPS was tested by immunoblotting (Table 1). LPS from the Western strains NCTC11637 and O:2 did not react with any of the MAbs. The former lost the O-polysaccharide chain, but the latter showed O-polysaccharide-containing LPS on SDS-PAGE (Figure 1). LPS from the Sydney strain reacted only with Le^y^ MAb, and LPS from 26695 reacted with the Le^x^ and Le^y^ MAbs. Among the Japanese strains, LPS from GU2 reacted with the Le^x^ and Le^a^ MAbs; LPS from DU1 reacted with the Le^x^, Le^y^ and Le^b^ MAbs; LPS from CA4 reacted with the Le^x^ and Le^a^ MAbs; LPS from CA5 reacted with the Le^x^ and Le^y^ MAbs; and LPS from CA2 reacted only with the Le^y^ MAb.

**Figure 2:**
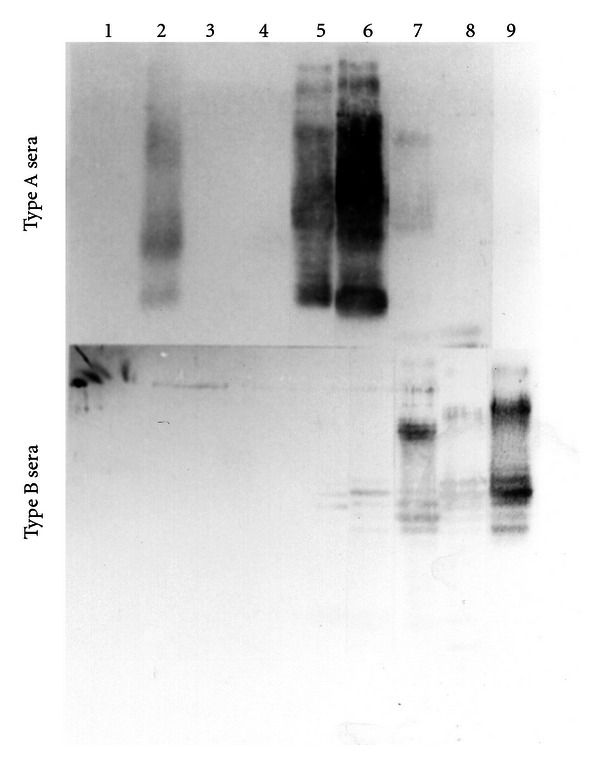
Immunoreactivities of two types of *H. pylori*-positive Japanese sera to *H. pylori *LPS from Japanese and Western strains. The LPS used are shown in Figure 1. Type A sera only reacted with LPS containing the highly antigenic epitope and Type B sera only reacted with LPS containing the weakly antigenic epitope. 1, NCTC11637-LPS; 2, Sydney strain-LPS; 3, 26695-LPS; 4, O:2-LPS, 5, GU2-LPS; 6, DU1-LPS; 7, CA2-LPS; 8, CA4-LPS; and 9, CA5-LPS.

 We examined the reactivities of sera from humans with *H. pylori* infection to *H. pylori* LPS by immunoblot analysis. We previously proposed in [7–9] the presence of two distinct epitopes, termed the highly antigenic and the weakly antigenic epitopes, on the O-polysaccharide chains, based on data from the immunoblotting of LPS with sera from *H. pylori*-positive humans. Furthermore, we also proposed the classification of *H. pylori*-positive human sera into three groups on the basis of immunoblot reactivity to the polysaccharide region of LPS [7–9]. Type A sera only react with LPS containing the highly antigenic epitope, type B sera only react with LPS containing the weakly antigenic epitope, and type C sera react with both types of LPS. Among the LPS from Western strains, LPS from the Sydney strain reacted with type A sera but not type B sera (Figure 2 and Table 1). The O-polysaccharides from the NCTC11637, 26695, and O:2 strains did not react with any of the human sera, while LPS from Japanese strains reacted with either type A sera or type B sera.

## 4. Discussion

 The O-polysaccharide regions of LPS are commonly used for typing gram-negative bacteria into O-serotype because of their high antigenicity, which is specific for each strain. In the case of *H. pylori* LPS, the properties of the epitopes of the polysaccharide region seem to be complex. It has been demonstrated chemically and immunogenically that the O-polysaccharide portions of *H. pylori* LPS contain structures that mimic the Lewis blood antigens [3, 4, 6, 13, 14]. Heneghan et al. [15] proposed that anti-Lewis antibodies were present in most patients with *H. pylori* infection and that this response is independent from the host Lewis phenotype but is related to the bacterial Lewis phenotype. However, Appelmelk et al. [16] suggested that the molecular similarity of the *H. pylori* LPS to the Lewis antigens did not promote immune evasion, nor does it lead to induction of autoantibodies. We also reported that, although high titers of antibodies to *H. pylori* LPS were found in the sera of infected patients, these antibodies were not autoreactive and were not directed against the Lewis antigens [6]. This discrepancy is interpreted to be due to differences in the Lewis antigen analogues used in the immunoanalysis.

 On the other hand, our earlier studies suggest that highly and weakly antigenic epitopes reacting with human sera are unlikely to be immunogenically related to the structures mimicking the Lewis antigens [7–9]. Interestingly, the expression of the two epitopes seems to be mutually exclusive; no strain expressing both epitopes was identified, but all smooth type strains isolated in Japan expressed one or the other [7]. In this study, we observed no reaction of type A or type B Japanese sera with smooth type LPS from the Western strains 26695 and O:2, while all LPS from Japanese strains and the Sydney strain reacted with the sera belonging to the type A or B. The Sydney strain seems to be close to the Asian strains, because this strain was isolated from long-term adapted mice after they were inoculated with a fresh homogenate of gastric biopsy specimen derived from an individual in Sydney, Australia [17]. The lines of evidence indicated that the LPS in the Western strains lacked the highly antigenic and the weakly antigenic epitopes that are shared by the Japanese strains.

## 5. Conclusion

The two Western *H. pylori* strains shared neither the highly antigenic epitope nor weakly-antigenic epitope, which are shared by all Japanese strains, in the O-polysaccharide region of LPS.

## Figures and Tables

**Table 1:** Reactivity of LPS from Japanese and Western *H. pylori* strains against anti-Lewis antigen monoclonal antibodies and *H. pylori*-positive sera from Japanese individuals.

LPS from	Silver stain	Reactivity of anti-Lewis antigen antibody				Reactivity of human sera	
		Le^x^	Le^y^	Le^a^	Le^b^	Type A^a^	Type B^b^
*Western strain*							
NCTC11637	Rough	−	−	−	−	−	−
Sydney	Smooth	−	+	−	−	+	−
26695	Smooth	+	+	−	−	−	−
O:2	Smooth	−	−	−	−	−	−
*Japanese strain*							
GU2	Smooth	+	−	+	−	+	−
DU1	Smooth	+	+	−	+	+	−
CA2	Smooth	−	+	−	−	−	+
CA4	Smooth	+	−	+	−	−	+
CA5	Smooth	+	+	−	−	−	+

^a^Type A sera only reacted with the highly antigenic epitope containing LPS;^b^TypeB sera only reacted with the weakly antigenic epitope containing LPS.
